# Emerging Bismuth Chalcogenides Based Nanodrugs for Cancer Radiotherapy

**DOI:** 10.3389/fphar.2022.844037

**Published:** 2022-02-18

**Authors:** Jia Huang, Qiong Huang, Min Liu, Qiaohui Chen, Kelong Ai

**Affiliations:** ^1^ Xiangya School of Pharmaceutical Sciences, Central South University, Changsha, China; ^2^ Hunan Provincial Key Laboratory of Cardiovascular Research, Xiangya School of Pharmaceutical Sciences, Central South University, Changsha, China; ^3^ Department of Pharmacy, Xiangya Hospital, Central South University, Changsha, China; ^4^ National Clinical Research Center for Geriatric Disorders, Xiangya Hospital, Central South University, Changsha, China

**Keywords:** bismuth chalcogenides, nanomaterials, cancer radiotherapy, radiosensitizers, tumor hypoxia microenvironment

## Abstract

Radiotherapy (RT), as one of the main methods of clinical tumor treatment, has been applied to the treatment of most solid tumors. However, the effect of RT is compromised by the radiation resistance of tumor hypoxic environment and non-specific damage caused by high-dose radiation. Bismuth chalcogenides (Bi_2_X_3_, *X* = S, Se) based nanodrugs have attracted widespread attention as highly efficient radiosensitizers due to their high photoelectric effect and excellent biocompatibility. More importantly, specially designed nanocomposites can effectively alleviate the radiation resistance of tumor tissues. Here, for the first time, we systematically summarize the latest progresses of Bi_2_X_3_ nanodrugs to enhance RT by alleviating the hypoxic tumor microenvironment. These emerging Bi_2_X_3_ nanodrugs mainly include three aspects, which are Bi_2_X_3_ nanocomposites with high-efficient O_2_ supply, non-O_2_-dependent Bi_2_X_3_ nanocomposites RT enhancers, and Bi_2_X_3_ nanocomposites-based photothermal-enhanced radiosensitizers. These Bi_2_X_3_ nanodrugs can effectively overcome the RT resistance of tumor hypoxic microenvironment, and have extremely high therapeutic effects and clinical application prospects. Finally, we put forward the challenges and prospects of Bi_2_X_3_ nanomaterials in the field of RT.

## Introduction

Radiotherapy (RT) has many advantages for cancer treatment compared with surgery or chemotherapy, like non-invasive, excellent targeting, and low cost (Begg et al., 2011). Currently, half of new cancers are treated with RT (Bentzen, 2006). RT adopts ionizing radiation (usually X-ray) to irradiate the tumor site through direct and indirect action to induce cancer cell death. Ionizing radiation can directly destroy DNA or protein by breaking the bonds in these molecules. More importantly, high-energy ionizing radiation can easily ionize and split H_2_O to produce many reactive oxygen species (ROS) in tumor tissues (Eq. 1) (Le Caër, 2011). These ROS further cause the death of cancer cells by damaging DNA and proteins (Wu et al., 2019; Yao et al., 2021a). This indirect effect is the main tumor-killing effect of RT because the water content is the highest (generally 65%) in tumor tissues. However, there are two bottlenecks which greatly limit the effectiveness of RT. Firstly, a larger dose of X-rays is usually required to kill tumor cells because cancer tissues absorb X-rays very weakly, which also cause damage to normal tissues, especially the immune system (De Martino et al., 2021). Secondly, the hypoxic tumor microenvironment (TME) greatly reduces the effect of RT. O_2_ is a very important RT sensitizer and is easy to accept a free electron to form superoxide radicals (O_2_^−^·), which is then further converted into other highly oxidative active ROS (e.g. hydrogen peroxide and hydroxyl radicals) (Zhao et al., 2022; Zhu et al., 2022). Compared with normal cells, cancer cells are 3-times more resistant to RT-induced killing in a tumor hypoxia environment (Evans et al., 1997).

$$H_{2}O\overset{Ionizing\;Radiation}{\to }e^{-}+\cdot OH+H\cdot +HO_{2}\cdot +H_{2}O_{2}+H_{3}O^{+}+HO^{-}$$ (1)

Drugs containing high atomic number elements can be very effective in enhancing RT because they have a much higher X-ray absorption capacity than human tissues. Currently, many kinds of elements with high atomic number have been researched for radiosensitization, such as Au, Ta, W, Yb, Hf, and Bi (Tang et al., 2019; Xie et al., 2019; Zang et al., 2019; Peng et al., 2020; Liu et al., 2021; Xue et al., 2021). For example, NBTXR3 based on HfO_2_ has been approved by the FDA to enter Phase Ⅲ clinical studies, and demonstrated excellent RT effect for advanced soft-tissue sarcoma (Bonvalot et al., 2019). However, most of the high-Z elements are heavy metal elements with high toxicity, and their application in the field of biomedicine has been greatly restricted.

**FIGURE 1:**
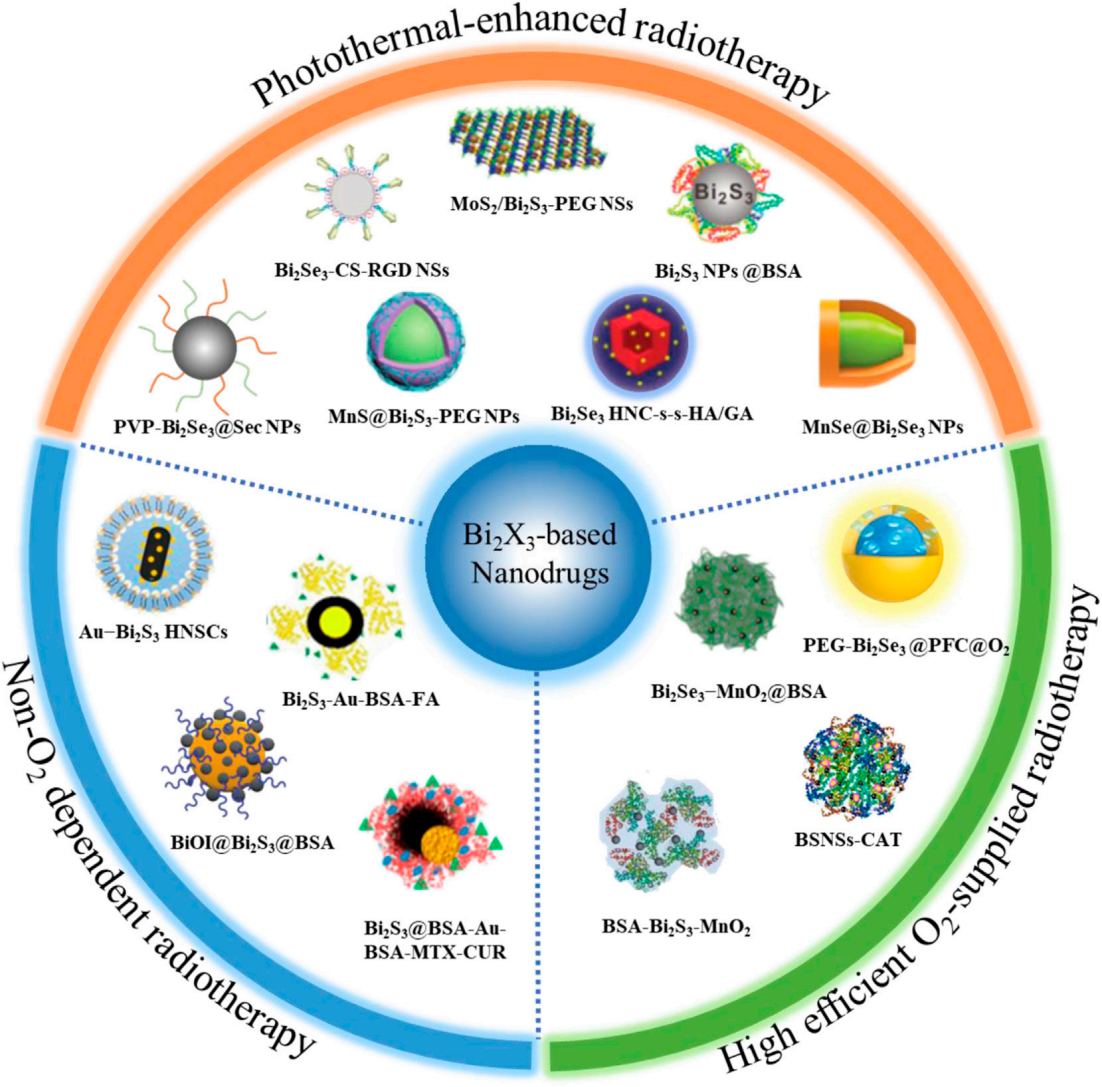
Overview of radiosensitization strategies based on Bi_2_X_3_ nanodrugs, which mainly including three aspects, the first is high efficient O_2_-supplied Bi_2_X_3_-based nanodrugs: such as perfluorocarbon-loaded hollow Bi_2_Se_3_ nanoparticles (PEG-Bi_2_Se_3_@PFC@O_2_ NPs), bismuth sulfide−albumin composite nanospheres followed by catalase conjugation (BSNSs-CAT), bovine serum albumin-coated Bi_2_S_3_ and MnO_2_ nanocomposites (BSA-Bi_2_S_3_-MnO_2_), bovine serum albumin-coated Bi_2_Se_3_ and MnO_2_ nanocomposites (Bi_2_Se_3_-MnO_2_@BSA); the second is Non-O_2_ dependent Bi_2_X_3_-based nanodrugs: such as Schottky-type heterostructure of Au-Bi_2_S_3_ (Au-Bi_2_S_3_ HNSCs), co-drug (MTX and CUR) loaded Bi_2_S_3_@BSA-Au semiconductor-metal heterojunction nanoparticles (Bi_2_S_3_@BSA-Au-BSA-MTX-CUR), folic acid (FA) functionalized and BSA-modified Bi_2_S_3_-Au heterodimers (Bi_2_S_3_-Au-BSA-FA); the third is photothermal-enhanced Bi_2_X_3_-based nanodrugs, such as PEGylated 2D MoS_2_/Bi_2_S_3_ composite nanosheets (MoS_2_/Bi_2_S_3_-PEG NSs), BSA-stabilized Bi_2_S_3_ Nanoparticles (Bi_2_S_3_ NPs@BSA), poly(vinylpyrollidone)-and selenocysteine-modified Bi_2_Se_3_ nanoparticles (PVP-Bi_2_Se_3_@Sec NPs), HA-functionalized gambogic acid (GA) loaded Bi_2_Se_3_ hollow nanocube (HNC-s-s-HA/GA) and MnSe@Bi_2_Se_3_ core–shell nanoparticles (MnSe@Bi_2_Se_3_ NPs).

Bismuth, as an element with high atomic number (*Z* = 83), has surprising biocompatibility and been active in the biomedical field for hundreds of years. A variety of bismuth-based compounds have been widely used to treat diseases such as gastrohelcoma and bacterial infections (Peterson et al., 1996; Nomiya et al., 2004). Bismuth chalcogenides (Bi_2_X_3_, *X* = S, Se) based nanodrugs have been favored in tumor RT due to their many unique characteristics: 1) low toxicity and high biological safety *in vivo*; 2) low cost and easy synthesis; 3) strong X-ray absorption (The X-ray attenuation coefficient of Bi element is 5.74 > Au = 5.16 > Pt = 4.99 > Ta = 4.3 cm^2^ g^−1^ at 100 keV). After Bi_2_X_3_-based nanodrugs specifically enrich in the tumor area by passively or actively targeting effect, the tumor can be effectively killed at a lower X-ray dose, and the damage to other normal tissues can also be greatly reduced (Zhang et al., 2014; Song et al., 2017; Alejo-Martinez et al., 2019). Nevertheless, the RT effect of these nanodrugs is still greatly reduced by the hypoxic tumor microenvironment. Currently, many emerging Bi_2_X_3_ nanodrugs are developed to further improve the efficiency of RT, and have demonstrated very impressive tumor-killing effects. Here, a systematic review is provided to summarize the breakthrough progresses of Bi_2_X_3_ nanodrugs for overcoming the limitations of the tumor hypoxia microenvironment in the field of RT. Currently, three strategies have been developed to improve the RT efficiency of Bi_2_X_3_ nanodrugs (Figure 1; Table 1). Firstly, elaborately designed Bi_2_X_3_-based nanocomposites increase the supply of O_2_ to relieve the hypoxic state of the TME; the second strategy is non-O_2_ dependent RT: Bi_2_X_3_-based nanocomposites with distinctive heterojunction structure to promote the production of non-O_2_ dependent radicals; the third is photothermal-enhanced RT: local high temperature of the tumor site can not only relieve the hypoxic tumor microenvironment, but also increase the yield and speed of ROS production in RT. Finally, we discussed the challenges and prospects of bismuth chalcogenides nanocomposites in the field of cancer RT.

**TABLE 1:** The overview of emerging Bi_2_X_3_-based nanodrugs for RT.

Category	Nanomaterials	Advantages of nanomaterials	Ref
High efficient O_2_-supplied radiotherapy	PEG-Bi_2_Se_3_@PFC@O_2_ NPs	Efficient oxygen carrying capacity; powerful radiosensitization performance	Song et al. (2016)
	BSA-Bi_2_S_3_-CAT NSs	Effective tumor homing and tumor hypoxia relief	Zhang et al. (2018)
	Bi_2_Se_3_-MnO_2_-BSA	Excellent CAT-like catalytic activity; high colloidal stability and biocompatibility	Yao et al. (2021b)
	BSA-Bi_2_S_3_-MnO_2_	Remarkable radiotherapeutic enhancement effect; without obvious toxic and side effects	Zhang et al. (2019)
Non-O_2_ dependent radiotherapy	Schottky-type heterostructure of Au-Bi_2_S_3_	Significant electron-hole separation efficiency, high-efficiency radiosensitization properties	Wang et al. (2019)
	Bi_2_S_3_@BSA-Au-BSA-MTX-CUR hybrid system	Efficient electron-hole separation efficiency and synergistic anti-tumor effects of radio-chemotherapy	Nosrati et al. (2022)
	Bi_2_S_3_-Au-BSA-FA hybrids	Effective radiosensitization and tumor targeting	Abhari et al. (2020)
Photothermal-enhanced radiotherapy	Bi_2_S_3_ nanorods	Remarkable radio-photothermal synergistic therapeutic effect	Cheng et al. (2017)
	BSA-capped Bi_2_S_3_ NPs	Ultra-small size; remarkable X-ray and photothermal response properties (η= 51%)	Wang et al. (2016)
	BSA-Bi_2_Se_3_ nanodots	High photothermal conversion efficiency (η= 50.7%); effective radiosensitization ratio (6%)	Mao et al. (2016)
	PVP-Bi_2_Se_3_@Sec NPs	Effective biodegradability; promoting the body’s immune function	Du et al. (2017)
	HA-functionalized gambogic acid (GA) loaded Bi_2_Se_3_ hollow nanocubes	Effective accumulation and uptake by CD44 overexpressing cancer cells; specifical drug releasing; avoiding heat damage	Song et al. (2019)
	(HNC-s-s-HA/GA)	—	
	Heterogeneous	satisfactory photothermal performance; enhanced radiosensitization effectively inhibit the TNBC metastasis	Fei Gao et al. (2020)
	Bi_2_S_3_-MoS_2_ NPs	—	
	MoS_2_/Bi_2_S_3_-PEG composite nanosheets	Desirable photothermal performance, colloidal stability and biocompatibility	Wang et al. (2015)
	Core–Shell MnSe@Bi_2_Se_3_-PEG	Additional MRI performance; photothermal-enhanced RT efficiency	Song et al. (2015)
	FeSe_2_/Bi_2_Se_3_-PEG composite nanostructures	Excellent compatibility, remarkable synergistic tumor destruction effect; no appreciable toxic side effect	Cheng et al. (2016)

### High-Efficient O_2_-Supplied Radiotherapy

Many well-designed Bi_2_X_3_-based nanocomposites have shown great potential in improving tumor hypoxia and RT efficiency. Bi_2_X_3_-based nanocomposites with ideal structure and morphology can be prepared through specific synthesis strategies due to their unique and flexible physical and chemical properties, such as hollow structure (Song et al., 2016; Zhang et al., 2020), mesoporous structure (Sun et al., 2019; Yang et al., 2021), core-shell structure (Li et al., 2017; Li et al., 2018). For example, Song et al. (2016) prepared PEGylated hollow Bi_2_Se_3_ nanoparticles (PEG-Bi_2_Se_3_ NPs) through cation exchange reaction based on the Kirkendall effect. Perfluorocarbon, a highly efficient oxygen loading solvent, was then filled into the hollow structure of PEG-Bi_2_Se_3_ NPs (PEG-Bi_2_Se_3_@PFC@O_2_). The oxygen carrying capacity of PEG-Bi_2_Se_3_@PFC@O_2_ was significantly higher than that of the hollow PEG-Bi_2_Se_3_ NPs, up to 96.9 ± 9.4 μmol/g of PEG-Bi_2_Se_3_. Moreover, the O_2_ retention time exceeded 1 h, and the gradual release of O_2_ effectively improved the hypoxic microenvironment in the tumor site. At the same X-ray dose, the anti-tumor effect of PEG-Bi_2_Se_3_@PFC@O_2_ was significantly better than that of PEG-Bi_2_Se_3_ and RT group. Another effective strategy to improve tumor hypoxia is to convert the high concentration H_2_O_2_ into O_2_ in the tumor microenvironment (Zhang et al., 2018; Zhang et al., 2019; Yuzhu Yao et al., 2021). For example, Zhang et al. (2018) developed a Bi_2_S_3_-albumin composite nanospheres combined with catalase (abbreviated as BSNSs-CAT) for cancer treatment. CAT at BSNSs-CAT efficiently catalyzed the conversion of H_2_O_2_ into O_2_ after BSNSs-CAT accumulated in tumor tissues through enhanced penetration and retention effect (EPR effect). The percentage of O_2_ saturation concentration treated with BSNSs-CAT increased significantly from 52.5% to about 59.2% in the tumor site. BSNSs-CAT + RT had the best tumor growth inhibition effect thanks to the strong reflective absorption of Bi and the improvement of the hypoxic microenvironment, followed by BSNSs + RT, then RT group. However, CAT, as a natural enzyme, is easily degraded and inactivated by proteases *in vivo*. Some catalase-mimick nanozymes can catalyze H_2_O_2_ to produce H_2_O and O_2_ (Dai et al., 2021). Very recently, Yuzhu Yao et al. (2021) developed a nanocomposite of Bi_2_Se_3_, MnO_2_ and bovine serum albumin (Bi_2_Se_3_-MnO_2_@BSA) for RT. MnO_2_ showed high-efficiency catalase-like properties and excellent stability *in vivo*. Moreover, the CAT activity of Bi_2_Se_3_-MnO_2_@BSA was 2.46 times higher than that of MnO_2_@BSA, because the Mn atoms of Bi_2_Se_3_-MnO_2_@BSA was in an electron-rich state and easier to provide electrons for H_2_O_2_. The Bi_2_Se_3_-MnO_2_@BSA + RT group showed a stronger tumor-killing effect compared to the MnO_2_@BSA + RT group and the RT group in the *in vivo* treatments.

### Non-O_2_ Dependent Radiotherapy

Non-O_2_ dependent RT has great advantages in RT, because it can directly avoid the RT resistance from the hypoxic microenvironment. As we all know, Bi chalcogenide compounds, as a narrow band gap semiconductor, can theoretically be excited by X-rays to generate free electrons and holes in the conduction band (CB) and valence band (VB), respectively (Meng et al., 2016; Waiskopf et al., 2016). These electron-hole pairs further react with H_2_O or H_2_O_2_ to generate highly cytotoxic hydroxyl radicals (·OH) to induce cancer cells apoptosis by intense oxidative damages. However, the generation of ·OH is significantly suppressed in Bi chalcogenide nanomaterials due to the rapid recombination of electron-hole pairs (Zhang et al., 2012). The heterojunction structure of Bi_2_X_3_ nanocomposites can separate electrons and holes to greatly reduce the recombination of electron and hole pairs (Wang et al., 2019; Abhari et al., 2020; Nosrati et al., 2022). For example, Wang et al. (2019) designed Au-Bi_2_S_3_ nanocomposites with Schottky-type heterostructures (Au-Bi_2_S_3_ HNSCs) for non-O_2_ dependent RT. Au-Bi_2_S_3_ HNSCs were prepared by *in-situ* growth of gold nanocrystals on Bi_2_S_3_ nanorods. The Schottky barrier was a low interface voltage region on the metal-semiconductor boundary. Semiconductor Bi_2_S_3_ generated low-energy electron-hole pairs under X-ray irradiation in Au-Bi_2_S_3_ HNSCs, and then electrons and holes were effectively separated because the electrons were easily transferred to gold *via* Schottky barrier. The current response of Au-Bi_2_S_3_ HNSCs was 1.5-times higher than pure Bi_2_S_3_ and the ·OH production was 1.6-times than that of Au and Bi_2_S_3_ mixture under X-ray irradiation. More importantly, the RT effect of Au-Bi_2_S_3_ HNSCs was significantly better than that of the pure Bi_2_S_3_ group or the Au and Bi_2_S_3_ mixture group both in the *in vitro* and *in vivo* experiments. In addition, Bi_2_S_3_-Au Schottky-type heterostructures can be adopted as a multifunctional drug delivery platform to combine chemotherapy and RT. This combination therapy has shown great potential in improving the efficiency of RT and minimizing the systemic toxicity of chemotherapeutic drugs (Nadar et al., 2021). Very recently, Nosrati et al. (2022) developed a methotrexate and curcumin co-loaded BSA-encapsulated Bi_2_S_3_-Au nanocomposite (Bi_2_S_3_@BSA-Au-BSA-MTX-CUR) for the combined treatment of chemotherapy and RT. In Bi_2_S_3_@BSA-Au-BSA-MTX-CUR, Bi_2_S_3_@BSA-Au heterojunctions enhance the generation of ·OH to increase the RT efficiency, while MTX efficiently promoted cellular uptake and interfere the biosynthesis of DNA of cancer cells. Interestingly, the combined treatment of chemotherapy and RT achieved a significant anti-cancer effect *in vivo* only under a single dose Bi_2_S_3_@BSA-Au-BSA-MTX-CUR injection and one-time X-ray irradiation, and the tumors was completely eradicated after 20 days of treatment.

### Photothermal-Enhanced Radiotherapy

In recent years, photothermal therapy (PTT), as a specific emerging cancer therapy, has been extensively researched in the field of tumor treatment (Liu et al., 2019; Danewalia and Singh, 2021). Many transition metal nanomaterials have been researched for PTT, such as MoS_2_-based nanomaterials (Jianling Wang et al., 2021), CoS_2_ nanomaterials (Wang et al., 2020a), copper-based nanomaterials (Ai et al., 2021; Wang et al., 2021b; Li et al., 2021), titanium-based nanomaterials (Wang et al., 2020b; Wang et al., 2021c), covalent organic frameworks (COFs) (Yao et al., 2021b), etc. Compared with above PTT agents, Bi_2_X_3_-based nanomaterials have been proven to be a kind of more excellent photosensitizers due to the strong near-infrared absorption performance and high photothermal conversion efficiency of Bi_2_X_3_ (Xie et al., 2016; Cheng et al., 2018). Local high temperature can directly increase the oxygen content of the tumor microenvironment by increasing blood flow in the tumor. Moreover, high temperature induced by PTT can facilitate the generation of O_2_-dependent ROS for RT by inhibiting the expression of hypoxia-inducible factor (HIF-1α) to increase oxygen concentration in tumor site. In addition, photothermal effects also interfere with DNA repair by reducing the expression of DNA repair related proteins (DNA repair enzymes, PARP, Rad 51), and downregulating angiogenic factors to inhibit tumor metastasis (Oei et al., 2015; Cheng et al., 2017). Therefore, the combination of photothermal therapy and RT is an effective radiosensitization strategy. For example, Wang et al. (2016) prepared ultra-small BSA-coated Bi_2_S_3_ nanodots (BSA-Bi_2_S_3_ NPs) for photothermal-enhanced RT. BSA-Bi_2_S_3_ NPs had the excellent X-ray and photothermal response properties (the photothermal conversion efficiency was as high as 51%). Moreover, The BSA-Bi_2_S_3_ NPs with ultra-small size (about only 6 nm) were more conducive to being taken up by tumor cells. Compared with the RT sensitization group (Bi_2_S_3_+X-ray) or the PTT group (Bi_2_S_3_+NIR), the 4T1-tumor bearing mice treated with radio-photothermal combination therapy group (Bi_2_S_3_+X-ray + NIR) achieved complete tumor eradication, and the survival rate of mice reached 100% over 40 days after treatment. In addition, it is also extremely important to protect adjacent normal tissues from radiation damage during RT. Recently, Du et al. (2017) reported a Bi_2_Se_3_ nanoparticles modified with polyvinylpyrrolidone and selenocysteine (PVP-Bi_2_Se_3_@Sec NPs) for photothermal-enhanced RT. The photothermal effect of Bi_2_Se_3_ NPs effectively improved tumor hypoxia microenvironment to enhance the radiosensitivity of cancer cells. Moreover, the PVP-Bi_2_Se_3_@Sec NPs were degraded *in vivo*, and part of the Se released from the NPs to enhance the body’s immune function. Compared with RT, the PVP-Bi_2_Se_3_@Sec NPs group effectively protected the immune system, and the key cytokines level (like interleukin 6 and 2) were restored in the blood.

The efficiency of RT can be further increased by improving the photothermal conversion efficiency of the Bi_2_X_3_-based nanocomposites. Fox example, Fei Gao et al. (2020) developed heterogeneous Bi_2_S_3_-MoS_2_ nanoparticles (BMNPs) for photothermal enhanced RT. BMNPs had a higher photothermal conversion efficiency than Bi_2_S_3_ nanoparticles (BNPs) (35.8 vs 28.1%). The BMNPs reduced the quasi-threshold X-ray dose from 1.39 to 0.92 Gy, and the sensitivity enhancement ratio increased by 17.9%. The effect of NIR + RT + BMNP group was much better than that of RT group and RT + BMNPs group in the treatment of triple-negative breast cancer. The survival rate of mice in the NIR + RT + BMNP group was as high as 100% at 28 days after treatment, while the RT group and RT + BMNPs group had only 0 and 20%, respectively. When the temperature of the tumor area rises, the tumor cells resisting heating-caused damage by up-regulating the expression of heat shock proteins (HSPs) (Ge Gao et al., 2020). Therefore, the photothermal enhanced RT can be further increased by inhibiting the activity of HSPs. Moreover, avoiding thermal damage and inflammation of adjacent normal tissues caused by hyperthermia also needs to be considered. Recently, Song et al. reported a hyaluronic acid (HA) modification and gambogic acid (GA) loaded hollow Bi_2_Se_3_ nanotube (HNC-ss-HA/GA) for low-temperature radio-photothermal combination therapy. HA ligands promoted the accumulation of HNC-ss-HA/GA in tumors due to its specifical affinity with CD44 receptor in cancer cells. Glutathione, one of the most important antioxidants in cells, is known to be overexpressed in cancer cells (Ding et al., 2021). Interestingly, the disulfide bond between HNC and HA can be rapidly cleaved by glutathione to release GA. GA, as an effective inhibitor of HSPs, which could enhance the heat sensitivity of cancer cells (Su et al., 2021), thereby improve the efficacy of photothermal-enhanced RT. The combined therapy group (HNC-s-s-HA/GA + NIR + X-ray) demonstrated the strongest suppress tumor growth effect *in vivo* compared to other monotherapy groups (HNC-s-s-HA/GA + NIR and HNC-s-s-HA/GA + X-ray).

## Summary and Outlook

In summary, this review summarizes the latest research progress of Bi_2_X_3_-based nanodrugs for RT. Bi_2_X_3_-based nanodrugs have great clinical application prospects in the field of RT because of their super-high RT effect and biocompatibility. Nevertheless, there are still many challenges to overcome in achieving clinical translation of these treatment strategies. Firstly, the excellent RT effects of these Bi_2_X_3_-based nanodrugs are all achieved in mice models. However, the huge species difference between human and mice makes these nanodrugs face a big bottleneck for clinical translation. For example, mice tumor models generally take about 15 days, while human cancers often take months or even years. Therefore, the tumor microenvironment of human may be very different from that of mice models, which may lead to unsatisfactory clinical effects of Bi_2_X_3_-based nanodrugs. Therefore, from the perspective of clinical application, it is necessary to verify the radiosensitizing effect of Bi_2_X_3_-based nanodrugs in humanized animal models, such as the monkey models. Secondly, metabolic pathway of Bi_2_X_3_-based nanodrugs needs further study *in vivo*. As we all know, as a heavy metal element, excessive Bi may cause some side effects such as renal toxicity, brain toxicity and neurological decline, which can be attributed to the tendency of Bi to bind to sulfhydryl groups in many important enzymes in the human body, resulting in the denaturation of enzymes and destroys its functionality. At present, most of the metabolism and toxicity of Bi_2_X_3_-based nanodrugs have only been done for about a month, and the longer-term toxicity and metabolic mechanisms still need to be further explored. Therefore, exploring biodegradable and clearable Bi_2_X_3_-based nanodrugs is of great significance for thier clinical translation (Wang et al., 2021a). Fortunately, there is rare Bi element in the human body itself. Therefore, the distribution, metabolism, and excretion process of Bi_2_X_3_-based nanodrugs can be easily tracked by the content and valence of Bi *in vivo*. Thirdly, the large-scale and controllable preparation of Bi_2_X_3_-based nanodrugs need to be further optimized. In commercial preparation, it is necessary to maintain precise control of the size, morphology, charge, and composition of nanomaterials to ensure uniformity and strict quality control. Therefore, exploring a simpler, faster, more precise and controllable synthesis process is vital for the clinical translation and commercial production of Bi_2_X_3_-based nanodrugs in the field of RT. Nevertheless, Bi_2_X_3_-based nanodrugs still have great clinical application prospects of RT. As mentioned earlier, NBTXR3 based on HfO_2_ have shown excellent effects in clinical phase III. In theory, Bi_2_X_3_-based nanodrugs have stronger biocompatibility and radiosensitization effect than HfO_2_ nanoparticles. We believe that Bi_2_X_3_-based nanodrugs will achieve true clinical RT treatment with the joint efforts of scientists from multiple disciplines such as chemistry, medicine, and biology in the near future.
